# Layer-by-layer ultraviolet assisted extrusion-based (UAE) bioprinting of hydrogel constructs with high aspect ratio for soft tissue engineering applications

**DOI:** 10.1371/journal.pone.0216776

**Published:** 2019-06-12

**Authors:** Pei Zhuang, Wei Long Ng, Jia An, Chee Kai Chua, Lay Poh Tan

**Affiliations:** 1 Singapore Centre for 3D Printing, School of Mechanical and Aerospace Engineering, Nanyang Technological University, Singapore, Singapore; 2 School of Materials Science and Engineering, Nanyang Technological University, Singapore, Singapore; 3 Engineering Product Development Pillar, Singapore University of Technology and Design, Singapore, Singapore; Michigan Technological University, UNITED STATES

## Abstract

One of the major challenges in the field of soft tissue engineering using bioprinting is fabricating complex tissue constructs with desired structure integrity and mechanical property. To accomplish such requirements, most of the reported works incorporated reinforcement materials such as poly(*ϵ*-caprolactone) (PCL) polymer within the 3D bioprinted constructs. Although this approach has made some progress in constructing soft tissue-engineered scaffolds, the mechanical compliance mismatch and long degradation period are not ideal for soft tissue engineering. Herein, we present a facile bioprinting strategy that combines the rapid extrusion-based bioprinting technique with an in-built ultraviolet (UV) curing system to facilitate the layer-by-layer UV curing of bioprinted photo-curable GelMA-based hydrogels to achieve soft yet stable cell-laden constructs with high aspect ratio for soft tissue engineering. GelMA is supplemented with a viscosity enhancer (gellan gum) to improve the bio-ink printability and shape fidelity while maintaining the biocompatibility before crosslinking via a layer-by-layer UV curing process. This approach could eventually fabricate soft tissue constructs with high aspect ratio (length to diameter) of ≥ 5. The effects of UV source on printing resolution and cell viability were also studied. As a proof-of-concept, small building units (3D lattice and tubular constructs) with high aspect ratio are fabricated. Furthermore, we have also demonstrated the ability to perform multi-material printing of tissue constructs with high aspect ratio along both the longitudinal and transverse directions for potential applications in tissue engineering of soft tissues. This layer-by-layer ultraviolet assisted extrusion-based (UAE) Bioprinting may provide a novel strategy to develop soft tissue constructs with desirable structure integrity.

## Introduction

The emerging 3D bioprinting approaches have offered a great potential in fabricating highly-complex, biomimetic tissue constructs by simultaneously depositing cells and biomaterials in a highly-specific arrangement ([1, 2]). The precise deposition of cells and biomaterials facilitates the important cell-cell ([3–5]) and cell-biomaterial interactions ([6–8]) for tissue maturation. Notably, designing a suitable microenvironment is highly critical for regulating cellular behavior ([9–12]). One of the major challenges in the field of soft tissue engineering is fabricating complex tissue constructs with compliant mechanical property and suitable structure integrity to avoid structural collapse ([13]). Most of the reported works incorporated reinforcement materials such as poly(*ϵ*-caprolactone) (PCL) polymer within the 3D bioprinted constructs to improve the mechanical stability ([14, 15]). However, the long period of degradation makes it less desirable for soft tissue engineering applications.

The different bioprinting approaches include extrusion-based ([16–19]), inkjet-based ([20, 21]), microvalve-based ([22, 23]), and laser-based systems ([24, 25]). Among these different approaches, extrusion-based bioprinting is the most prevalent approach due to its fast fabrication speed, ease of operation and compatibility with various bio-inks ([17]). An ideal bio-ink should exhibit good printability, biocompatibility and compliant tissue stiffness ([26–29]). Most of the existing bio-inks are modified from natural biomaterials such as gelatin ([30–34]) and collagen ([35–37]) to form new composite bio-inks with tunable properties. Particularly, gelatin methacryloyl (GelMA) has been identified as a promising bio-ink owing to its excellent biological properties and tunable physical properties ([38, 39]). GelMA-based bio-inks have been utilized in the field of tissue engineering and regenerative medicine, such as cartilage ([40]), neural tissues ([41]), cardiac tissues ([42, 43])and even musculoskeletal tissues ([44]). However, it is important to note that high GelMA concentrations (≥ 10%) usually result in limited cell activity due to the relatively high crosslinking density and stiffness of the photo-crosslinked constructs ([45]), while low GelMA concentrations lead to poor printability with low printing resolution and poor shape fidelity. Moreover, the GelMA-based bio-ink has a narrow printing process window which is highly dependent on the printing temperature. Hence, further optimization is required to improve the stability and printability of GelMA bio-inks. A plethora of methods have been explored to improve the rheological behavior of GelMA, such as the addition of various materials like nanosilicates ([46]), partial crosslinking GelMA with enzymes ([33]), or through cooling process ([45]). Among these methods, gellan gum, which is a non-toxic polysaccharide, has been discovered as a promising rheological modifier to improve the rheological property of the bio-ink ([40, 47–49]).

Although GelMA-gellan gum (GelMA-GG) bio-ink has exhibited great potential in improving the printabiliy of GelMA-based bioink, the excessive addition of gellan gum may in turn compromise the biocompatibility. Hence, minimum ideal amount of gellan gum in GelMA-based bioinks was selected to endow the enhanced printability of GelMA-GG and balanced biocompatibility. Meanwhile, layer-by-layer UAE bioprinting was implemented to reinforce the structure stability and printing resolution when constructing thick cell-laden tissue constructs. As such, we have demonstrated the ability to fabricate bioprinted constructs with high aspect ratio via a layer-by-layer UAE bioprinting strategy. Specifically, 30 different combinations of GelMA-GG bio-inks were investigated systematically through 3 main stages: 1) bio-ink preparation phase, 2) printing phase and 3) post-printing phase. The cells were first loaded in the composite bio-inks to evaluate its ease of cell encapsulation and potential cell sedimentation by analyzing the cell distribution within the composite GelMA-GG bio-inks. Next, the selected bio-inks were printed to evaluate its printability and printing accuracy of the 3D-bioprinted constructs through the UAE printing method. The printing conditions were optimized by adjusting the printing parameters to achieve structures with high aspect ratio. The UV effects on printing resolution and cell behavior have been investigated. Lastly, the 3D printed constructs were evaluated in terms of their material properties (material microstructure and compressive modulus) and corresponding cell behavior within the 3D-bioprinted cell-laden constructs. The study offers a new bioprinting strategy to generate stable 3D structures with compliant mechanical property and high aspect ratio using GelMA-based (GelMA-GG) bio-inks for engineering of soft tissue constructs.

## Materials and methods

### Preparation of GelMA-GG bio-inks

GelMA was prepared by reacting 10% (w/v) gelatin (Sigma Aldrich, type A from porcine skin, 300g Bloom, Singapore) with methacrylic anhydride (Sigma Aldrich) at 50°C as previous research described ([50]). The solution was dialyzed in 12-14kDa dialysis tubing (Sigma Aldrich) against distilled water at 40°C for 7 days, followed by 7-day lyophilization. It was then stored at -30°C for future use. Irgacure 2959 (Sigma Aldrich) was dissolved in 10% PBS (v/v) at 70°C to achieve the final concentration 0.1% (w/v) as described before ([48]). 6% sucrose (Sigma Aldrich) was added to generate an isotonic solution. GelMA was added into the PBS-based reagent at room temperature in varying quantities to achieve the concentrations at 2%, 4%, 10%, 15%, 20%, 30% (w/v) stored in 37°C incubator for future use. Low acyl gellan gum (Gelzan CM, Gelrite) was purchased from Sigma Aldrich. Gellan gum solution was prepared by dissolving gellan gum powder in the PBS-based solution to concentrations at 0%, 0.2%, 0.4%, 1%, 1.5% (w/v). The GelMA-GG composite bio-inks were formed by mixing GelMA and gellan gum solution at 45°C in 1:1 ratio for 1h to achieve 30 different combinations of GelMA-GG composite bio-inks. GelMA-GG with varied concentrations were recorded with their final concentrations in the form of weight over volume (w/v). For instance, 7.5-0.2 indicated that the GelMA-GG comprises 7.5%GelMA and 0.2% (w/v) GG.

### Evaluation of rheological properties of the GelMA-GG bio-ink

The rheological properties of the different composite bio-inks were tested using a rheometer (MCR 501, Anton Paar Germany GmbH, Ostfildern, Germany). The viscosity and the shear thinning properties of the different composite bio-inks were evaluated by using a cone plate (angle:1°) with 25 mm diameter. The bio-ink viscosities were evaluated for shear rates ranging from 0.1 to 500 s^−1^ at both 37°C (cell encapsulation temperature) and 25°C (printing temperature) to evaluate the suitable range of bio-ink viscosities for cell encapsulation and bioprinting process respectively. All measurements were performed in triplicate.

### Cell encapsulation and sedimentation

C2C12 (ATCC CFL-1772 Mus musculus muscle) rat myoblasts were labeled fluorescently with CellTracker Green CMFDA (Thermo Fisher) and then gently mixed with GelMA-GG bio-inks at 37°C to achieve a final cell density of 4×10^6^ cells/ml. The cell-laden bio-inks were loaded into printing cartridges and then were kept at 25°C (emulating printing temperature) for 1.5 hours to evaluate the effect of cell sedimentation. The GelMA-GG composite bio-inks were then frozen in 4°C for 20 mins after 1.5 hours of encapsulation to fix the cells under physical crosslinking for imaging. The cell-laden bio-inks were carefully observed using an inverted microscope (Carl Zeiss Axio Vert. A1).

### UV effects on printing resolution and cell viability

After determining the suitable bio-inks with homogeneous cell distribution through the cell encapsulation and sedimentation study, the printing study was performed on those bio-inks (GelMA-GG) at a printing temperature of 25 ± 1°C using a bioprinter (Regenhu, Villaz-St-Pierre, Switzerland). The printing process was then performed to evaluate the bio-ink printability and their printing resolution using 27G (210 *μ*m inner diameter) needle. To optimize the printing process, 2D structures were first printed to determine the bio-ink printability and then further tests were conducted to determine the optimal printing pressure and feed rate. Additionally, UV scanning speed has shown critical effects on printing resolution and cell viability. To study the influence of UV scanning speed on printing resolution, C2C12 encapsulated GelMA-GG constructs with 1, 3, 5, 7, 9 and 11 layers were printed into grid pattern under fixed printing speed and pressure. The width of the printed filaments were measured to determine the change of printing resolution overtime. Meanwhile, Live/dead staining and ImageJ was used to analyse the cell viability in the printed constructs. Cells in the bottom layer of the constructs were by imaged by fluorescent microscope to determine the UV influence on cell viability by cell counting analysis. After which, 3D grid and tubular constructs of different aspect ratio at varying diameter were printed with layer-by-layer UV curing approach using the identified optimal printing parameters with all the printable bio-inks.

### Evaluation of bio-ink’s mechanical properties (cyclic compression test)

The mechanical properties of the printed GelMA-GG bio-inks were tested with a uniaxial compression tester (Instron 5569, UK) at room temperature with 100 N load cell. All the samples were prepared into a cylindrical shape with a diameter of 12 mm and 5 mm height. The cyclic tests were recorded over 5 cycles at 30% strains, followed by continual compression at a rate of 2 mm/min until failure. The Young’s modulus was calculated as the slope of the linear region of the stress-strain curve in the 0-10% of the strain range.

### Evaluation of bio-ink’s microstructure (FE-SEM imaging)

Field Emission Scanning Electron Microscope (FE-SEM) imaging was carried out to analyze the microstructures of the printed GelMA-GG constructs. GelMA-GG constructs were printed in a rectangular shape (10 mm × 10 mm × 2 mm) and were dehydrated using graded ethanol (starting from 25, 50, 75, 90, 95 to 100% v/v). The samples were then dried using a critical point dryer (Leica EM CPD030, Germany) to retain the microstructure within the printed GelMA-GG constructs. The dried samples were then carefully sectioned using a sterile surgical blade to expose the cross-section before coating the samples with platinum (Pt) using a sputtering machine (Polarin SC7640 Sputter Coater from Quorum Technologies, United Kingdom). Representative images of GelMA-GG microstructure (n = 6) were taken at a 30,000x magnification using Ultra-Plus FE-SEM (Carl Zeiss, Germany). ImageJ was used to analyze the FE-SEM images to determine the pore sizes and porosity within the GelMA-GG microstructures at varying concentrations.

### Manual casting of cell-laden bio-inks

Immortalized C2C12 mouse myoblast cells were cultured in DMEM with 10% fetal bovine serum (FBS, Gibco), 1% antibiotic-antimycotic (HyClone) in 37°C, 5% CO_2_ environment. The culture medium was changed every 2∼3 days and the cells were harvested at 60-70% confluency. C2C12 cells were gently mixed with GelMA-GG bio-inks (5-0.5, 7.5-0.1, 7.5-0.2, 7.5-0.5, 10-0.1, 10-0.2) to a final cell density of 4×10^6^cells/ml. The cell-encapsulated bio-inks were then casted into pre-designed square PDMS mold with a dimension of 10 × 10 × 1 mm, followed by UV crosslinking. Live/Dead staining was performed on Day 0, 7 and 14. Live/dead staining was performed using Molecular Probes Live/Dead staining kits (Life-Technologies). The calcein AM will stain the viable cells green, while the ethidium homodimer-1 will stain the dead cells red. The samples were washed thrice with 1× PBS and 1 mL of staining solution was added to each of the 12-well plates containing the GelMA-GG bio-inks and was incubated for 30 mins at room temperature before observation under Inverted Microscopy (Carl Zeiss Axio Vert. A1). This study allowed us to identify suitable bio-inks for cell proliferation and spreading prior to actual printing.

### 3D bioprinting of cell-laden bio-inks

C2C12 cells were printed using a bioprinter (Regenhu, Villaz-St-Pierre, Switzerland) to study the influence of material stiffness and microstructure on the cell behaviour. Based on the earlier study of manual casting cell-laden bio-inks, we have selected a single bio-ink from each group to represent bio-inks of different material stiffness. The bio-inks were first printed using the extrusion-based print-heads, followed by curing each subsequent layer of printed bio-ink with a built-in UV-lamp (150mW, 365 nm wavelength). All the printing cartridges, needle tips, pyrex bottles and sucrose solution were fully autoclaved before use. C2C12 cells were first suspended in an isotonic sucrose solution at 37°C, followed by gently mixing with GelMA-GG bio-inks at 37°C using a pipette (Gilson) before transferring the cell-laden bio-inks into the sterilized Nordson printing cartridge. The 3D constructs were printed at 25 ± 1°C using a 27G needle tip (inner diameter: 210 *μ*m). The printed constructs were cultured over a week; live/dead staining and Prestoblue proliferative assay were performed on Day 1,3,7 to evaluate the cell viability and proliferation rates. Live/Dead staining was performed using Molecular Probes Live/Dead staining kits (Life-Technologies). The calcein AM will stain the viable cells green, while the ethidium homodimer-1 will stain the dead cells red. The samples were washed three times with PBS and 1 mL of staining solution was added to each of the 12-well plates containing the samples and incubated for 30 min at room temperature before observation under Inverted Microscopy (Carl Zeiss Axio Vert. A1). Cell proliferation was examined with Prestoblue assay. PrestoBlue reagent is quickly reduced by metabolically active cells, offering a quantitative measure of cell viability. Briefly, at each time point, sample in each well was cultured with 360 *μ*l medium plus 40 *μ*l presto blue incubated for 2hrs at 37°C. Standard test (control) was performed in parallel. A series of cells with different numbers (0, 10000, 20000, 50000, 75000, 100000, 125000, 150000, 175000, 200000, 300000) were seeded in 12-well plates and culture for 2hrs, then the medium was removed, and cells were cultured with 360*μ*l and 40*μ*l prestoblue for another 2hrs. Thereafter, aliquots were pipetted into a new 96-well plate with 100*μ*l for each well. The 96-well plates were then placed into a microplate reader and fluorescent mode was used to measure. The excitation at 560 nm and emission at 590 nm of the content of each well was measured. A standard calibration curve was generated from the control group, which was used to determine their corresponding background absorbance and these values were subtracted from the measurements. By comparing the extracted value from the samples to standard ones, cell numbers in each well could be calculated.

### Statistical analysis

All results were expressed as (*mean value* ± *standard deviation*(*SD*)). The results were evaluated by one-way ANOVA analysis coupled with the Tukey test. Differences are considered statistically significant when p ≤ 0.05 and greatly significant when p ≤ 0.001. All experiments were performed in triplicate.

## Results and discussion

**Fig 1:**
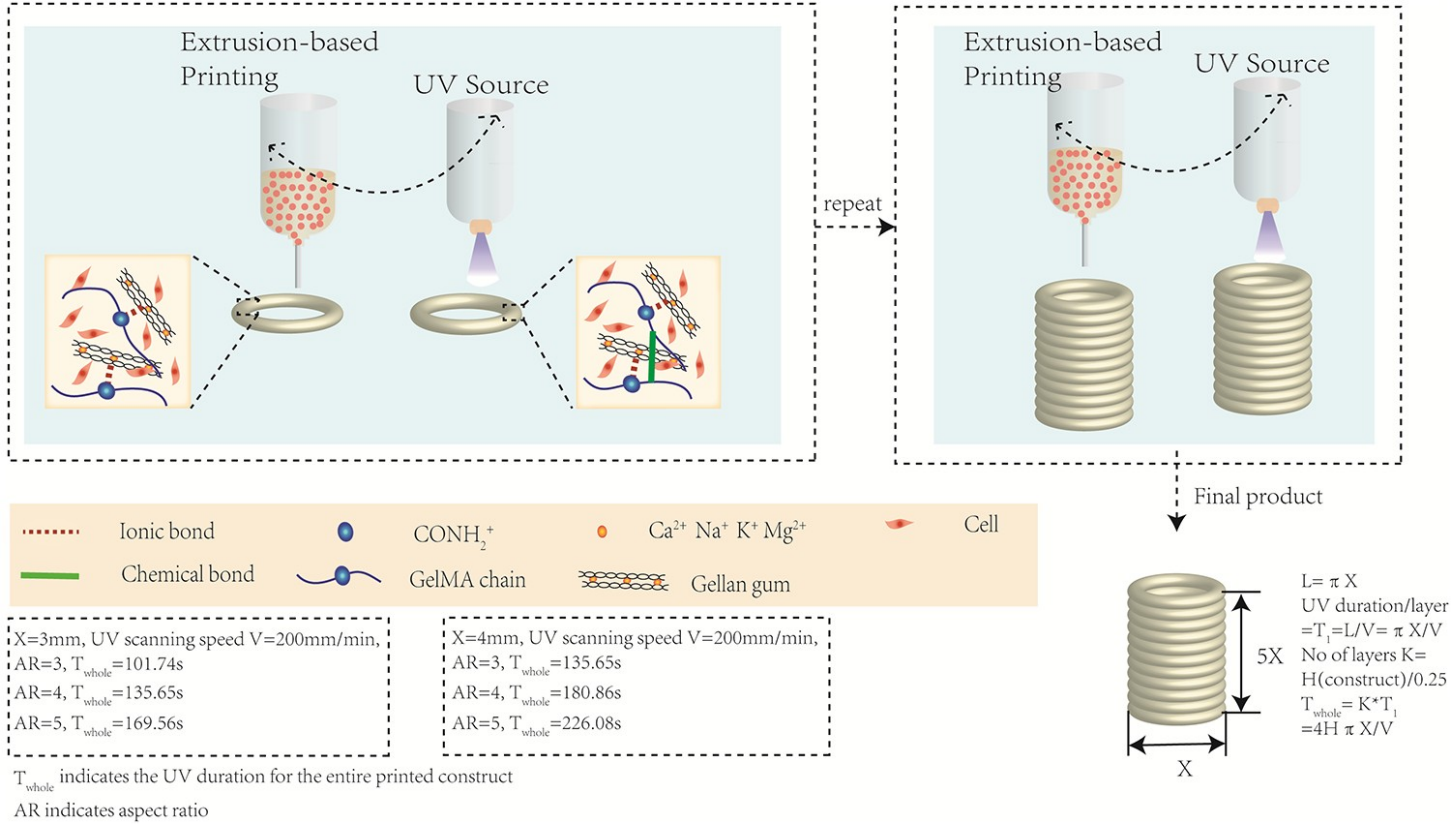
Schematic drawing of layer-by-layer UV-assisted bioprinting strategy. The gellam gun in the bio-ink serves as a viscosity enhancer to improve the bio-ink printability (via formation of ionic bonds between GelMA chain and gellan gum) during the extrusion printing process prior to further UV crosslinking (to form chemical bond between adjacent GelMA chains) of each individual printed layer. This layer-by-layer UV-assisted bioprinting strategy is repeated to eventually achieve fabrication of complex 3D structures with high aspect ratio.

The stringent requirements of bio-inks have resulted in the limited choice of printable cell-laden bio-inks. Some biocompatible materials (e.g collagen and hyaluronic acid) have exhibited poor printing resolution and weak mechanical property that constantly lead to structural collapse. Particularly, for soft tissues, it is difficult to find a suitable material with compliant mechanical property of native tissues while maintaining good structural integrity after several layers of printing. In contrast, GelMA is a thermo-sensitive material with highly tunable mechanical stiffness and has been commonly used for various biomedical applications due to its suitable biological properties and tunable physical characteristics. GelMA-based bio-inks possess important properties of native extracellular matrix (ECM) due to the presence of arginylglycylaspartic acid (RGD) peptide motifs that favor cell attachment, spreading and proliferation. However, the poor rheological properties of GelMA bio-inks at low concentrations (<10%) have resulted in poor printability and instable structures. Previous studies have reported GelMA or gelatin printing through bioprinters that are equipped with a temperature control system ([51]). However, most commercial bioprinters do not usually include such a temperature control system, hence posing a challenge to maintain a consistent printability of GelMA-based bio-inks due to fluctuations in temperature during printing process. Gellan gum is a thermo-reversible water-soluble anionic polysaccharide which has been used to improve the bio-ink printability by modifying the viscosity ([49]). Gellan gum (GG) is used to reinforce GelMA, leading to a more robust composite hydrogel. However, the printability of GelMA-GG is still limited to simple and relatively thin structures. Herein, we introduce a bioprinting strategy that enables the fabrication of tissue constructs with high aspect ratio via a layer-by-layer UAE bioprinting strategy (Fig 1).

### Bio-ink preparation

**Fig 2:**
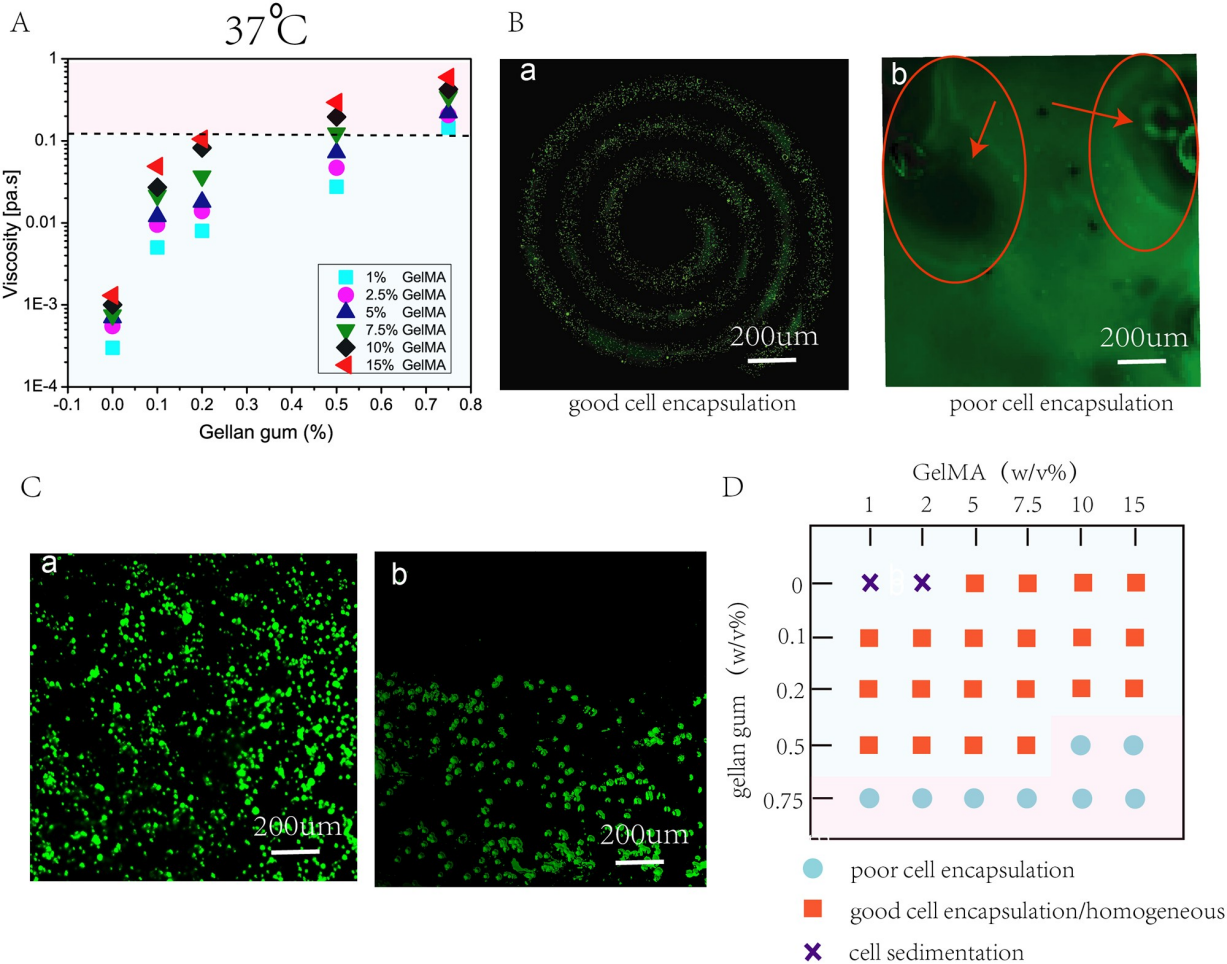
Bio-ink formulation involves characterization of rheological properties, ease of cell encapsulation and influence of cell sedimentation within bio-inks. A) Viscosity of 30 different GelMA-GG bio-inks at a constant shear rate of 100s^−1^ at 37°C indicate higher bio-ink viscosity with increasing polymer concentrations. B) Representative images to highlight the influence of bio-ink viscosity on cell encapsulation; a low viscous bio-ink facilitates good cell encapsulation (in spiral pattern with 7.5-0.2% w/v GelMA-GG) whereas a highly viscous bio-inks results in poor cell encapsulation. C) Representative images to highlight the influence of bio-ink viscosity and density on cell sedimentation; a low polymer concentration (low viscosity and density) and without gellan gum leads to cell sedimentation whereas a high polymer concentration (high viscosity and density) results in a homogeneous cell distribution with negligible cell sedimentation. D) An overview of the different GelMA-GG bio-inks in terms of cell encapsulation and cell distribution.

Bio-ink viscosity plays an important factor influencing cell encapsulation within bio-inks. To evaluate the quality of cell encapsulation, rheological behavior of GelMA-GG was investigated at 37°C (incubation temperature) (Fig 2A). The rheological behavior of the bio-ink was manipulated by varying the concentrations of GelMA and gellan gum.

A significant increase in the bio-ink viscosity (as shown in Fig 2A) at a constant shear rate of 100s^−1^ at 37°C (emulating encapsulation process) was observed across all GelMA concentrations with increasing gellan gum concentration from 0.1 to 0.75%. In contrast, a less significant increase in the bio-ink viscosity was observed when the GelMA concentration was varied from 1 to 15%. During the cell encapsulation process, cell pellets of desired cell density were pipetted into the bio-inks and mixed manually. Generally, homogenous cell distribution was observed in most of the GelMA-GG bio-inks in this study. However, the results have shown that the addition of 0.75% GG in all GelMA-GG bio-inks resulted in the formation of highly viscous bio-inks at 37°C which was inappropriate for cell encapsulation. Furthermore, GelMA-GG bio-inks of 10-0.5% w/v (∼0.2 Pa·s) and 15-0.5% w/v (∼0.3 Pa·s) also exhibited poor cell encapsulation. The representative images of the encapsulated cells in the GelMA-GG bio-inks are shown in Fig 2B; homogeneous cell encapsulation was observed in low viscous bio-inks, spiral pattern was printed to show the homogeneous cell distribution (Fig 2Ba) while non-homogeneous cell distribution with trapped air bubbles was spotted in highly viscous bio-inks (Fig 2Bb). The low viscous bio-inks (less than 124 mPa·s) facilitated easy cell encapsulation and resulted in homogeneous cell distribution within the cell-encapsulated bio-inks (left of Fig 2B), whereas the highly viscous bio-inks (over 124 mPa·s) are generally considered to be unsuitable for cell encapsulation due to the relatively higher bio-ink viscosities that led to non-homogeneous cell distribution and trapped air bubbles (Fig 2Bb). As such, our study has shown that a bio-ink viscosity of ∼0.15 Pa·s could be the threshold bio-ink viscosity for homogeneous cell encapsulation at 37°C.

Another important consideration is the cell sedimentation effect within the bio-inks over time. An ideal cell-laden bio-ink should result in a homogeneous cell output over time by mitigating the effect of cell sedimentation during the printing process. As such, we further investigated the effect of cell sedimentation in the cell-laden bio-inks with homogeneous cell distribution over a period of 1.5 hours (typical printing duration for large tissue constructs). All the selected GelMA-GG bio-inks were mixed with fluorescently-labelled cells and were evaluated over a period of 1.5 hours to study the effect of cell sedimentation which is caused by the gravitational forces acting upon the encapsulated cells within the bio-inks. The GelMA-GG composite bio-inks were frozen in 4°C for 20 mins after 1.5 hours of encapsulation to fix the cells under physical crosslinking at 4°C for imaging and are carefully observed using an inverted microscope (Carl Zeiss Axio Vert. A1). It was observed that there was negligible cell sedimentation effect in most of the GelMA-GG bio-inks over a period of 1.5 hours. The density and viscosity of the bio-inks increase with increasing polymer concentration; which explains the negligible cell sedimentation effect found in most of the GelMA-GG bio-inks (Fig 2Ca). In contrast, significant cell sedimentation effect was observed in 1-0% w/v and 2-0% w/v bio-inks (low GelMA concentration and without gellan gum) (Fig 2Cb). Particularly, the 1-0% w/v and 2-0% w/v GelMA-GG bio-inks still remained in liquid state whereas the other bio-inks have transited into a gel state after 1.5 hours of encapsulation. The low viscosity and density of 1-0% w/v and 2-0% w/v bio-inks led to significant cell sedimentation, hence they were not suitable to be used as cell-laden bio-inks (Fig 2D).

### Printing phase

**Fig 3:**
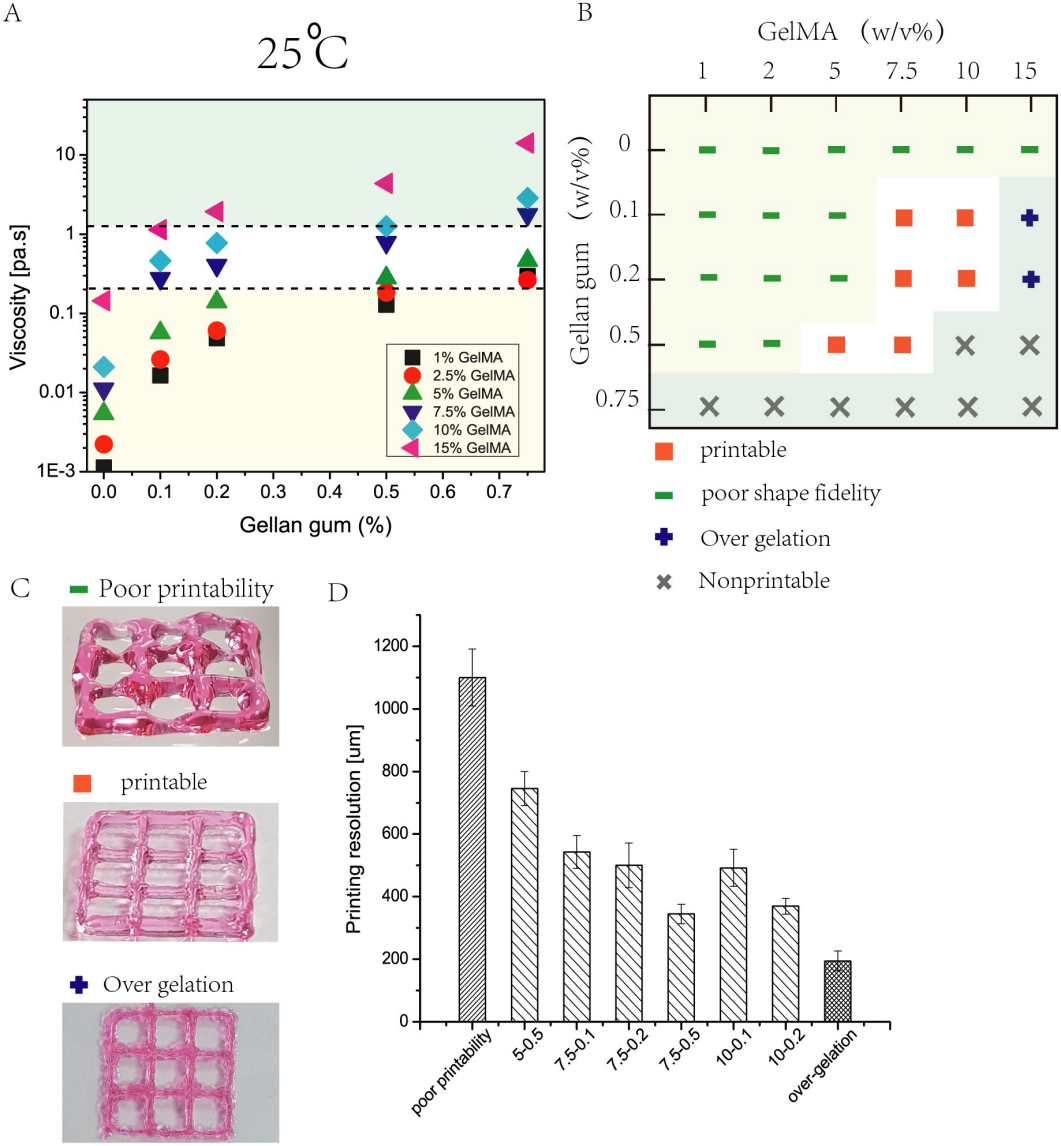
The bioprinting phase involves characterization of rheological properties, determination of suitable UV scanning speed and selection of suitable bio-inks. A) Rheological properties of 30 different GelMA-GG bio-inks at a constant shear rate of 100s^−1^ at 25°C indicated higher bio-ink viscosity with increasing polymer concentrations. B) An overview of the different GelMA-GG bio-inks in terms of printability and cell encapsulation. C) Representative images of printed constructs to distinguish among the three different classifications; (Top) poor printability, (Middle) good printability, (Bottom) over-gelation. D) Influence of bio-ink on printing resolution, a more viscous bio-ink results in higher printing resolution due to significantly less spreading of the shear-thinning bio-inks upon contact with the substrate surface.

The rheological properties of the composite bio-inks exhibited shear thinning behavior which was desirable for bioprinting applications. The rheological properties of the bio-inks were conducted at 25°C to evaluate the bio-ink viscosity at printing temperature (25 ± 1°C). Fig 3A presented the bio-ink viscosities at a fixed shear rate of 100 s^−1^ for all the groups. Generally, the bio-ink viscosity increased with increasing polymer concentration. As the GG concentration increased up to 0.75%, the viscosity increased significantly across all the GelMA concentrations. Similarly, GelMA concentration also exhibited the same trend albeit less dramatically as GG. A high viscosity within the printable range helped to reduce bio-ink spreading upon contact with the substrate surface prior to UV crosslinking.

To understand the correlation between the bio-ink viscosity and printability, different combinations of GelMA-GG bio-inks were used to print 2D filaments as a preliminary study to evaluate the bio-ink printability. The bio-ink printability can be classified into 3 different groups: 1) poor printability (Fig 3C Top), 2) good printability (Fig 3C Middle) and 3) over-gelation (Fig 3C Bottom). Poor printability is defined by the poor structural fidelity of the 3D printed constructs and inability to stack 3D constructs, whereas over-gelation refers to the excessive printing pressure required to extrude the bio-inks through a printing nozzle. Good printability is defined by the ability to print straight filament and 3D constructs using a moderate printing pressure (less than 3 bars ([52]). Although the GelMA-GG bio-inks were printed using the same nozzle diameter of 210 *μ*m, it is important to note that higher printing pressure was required to dispense the more viscous bio-inks. Printing resolution generally improved with increasing bio-ink viscosity as shown in Fig 3D. GelMA-GG bio-ink of 5-0.5% (w/v) showed a printing resolution of 745.6 ± 54.2 *μ*m at 0.8 bars printing pressure while GelMA-GG bio-ink of 10-0.2% w/v showed a printing resolution of 369.6 ± 25.1 *μ*m at 2.0 bars printing pressure. Particularly, the GelMA-GG bio-ink of 7.5-0.5% w/v (with the highest printable viscosity) exhibited the highest printing resolution of 344.6 ± 30.9 *μ*m at 1.5 bars printing pressure. The bio-inks with good printability were highlighted in Fig 3A within the white window; 6 combinations of GelMA-GG bio-inks in Fig 3B (5-0.5%, 7.5-0.1%, 7.5-0.2%, 7.5-0.5%, 10-0.1% and 10-0.2% w/v) showed good cell encapsulation, negligible cell sedimentation and good printability.

### UV effects on printing process and cell viability

**Fig 4:**
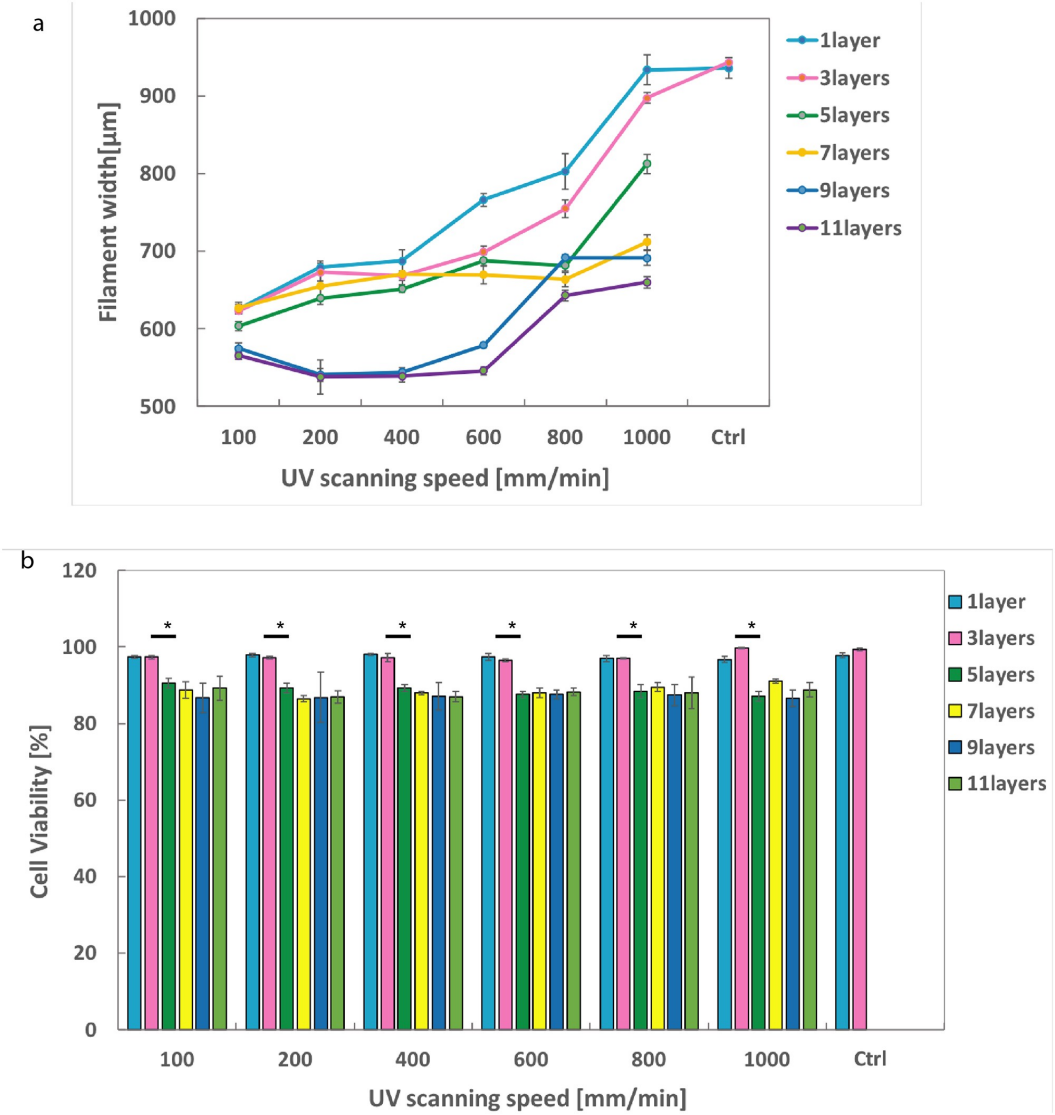
a) The effects of UV scanning speed (100, 200, 400, 600, 800, 1000 mm/min) on filaments width. b) The effects of UV scanning speed (100, 200, 400, 600, 800, 1000 mm/min) on cell viability. Constructs with no UV curing were used as control group. *—p < 0.05, **—p < 0.001.

As the layer-by-layer UAE bioprinting method utilizes UV source (365nm wavelength,mJ/cm^2^) to stablize the printed constructs, the critical effects of UV on printing resolution and cell behaviors should be examined. Optimization of the UV scanning speed was performed at different UV scanning speed (100, 200, 400, 600, 800, 1000 mm/min, no UV) and the resultant printing resolution was monitored. 7.5-0.2 GelMA-GG was employed and the printing speed was fixed at 100mm/min under 1.2 bar pressure with 250*u*m layer thickness for each layer. Printing resolution was monitored by measuring the width of the printed ring pattern (1, 3, 5, 7, 9 and 11 layers) with thinner filaments indicating higher printing resolution. The results in Fig 4a show that, a slower UV scanning speed led to a higher printing resolution (from 935.50 ± 13.29 *μ*m for control group without UV to 933.44 ± 18.95 *μ*m at 1000mm/min to 625.37 ± 6.08 *μ*m at 100mm/min) for a 1-layer construct. As the number of printing layers increased, the effect of UV scanning speed on the filament width seem to diminish. It can be seen that the increase in filament width with increasing UV scanning speed to be at the most prominent when printing 1 and 3 layers. The results indicated that under a fixed printing speed and printing pressrue, a slower UV scanning speed led to a longer UV crosslinking duration which resulted in higher degree of UV crosslinking and consequently reduced bio-ink spreading when subsequent layers were printed directly over it. Hence, filament width in each layer tends to be more stable and consequently improving the overall printing resolution and structural fidelity. Structure collapse occurred in control group after deposition of 3 layers due to insufficent self-support capability without UV crosslinking.

Additionally, it is also critical to ensure a suitable UV radiation duration that not only achieves adequate crosslinking but also high cell viability. In this work, the influence of UV on cell viability was characterized instantly post-printing by evaluating the cells at the botton-most layer of the constructs using live/dead staining kit. The cells at the bottom most layer would be those that had been subjected to the longest UV exposure and most prone to cell death caused by UV exposure, if any. The results in Fig 4b demonstrated that for 1-layer and 3-layer constructs, cell viability maintained above 95%. Starting from layer 5, cell viability showed a slightly drop but still remained above 85%. And the difference of cell viability among the groups was not statistically significant. From 5 layers onwards, cell viability remained relatively stable, indicated that the UV penetration depth is around 5 layers, which equals to 1.25mm. Taken together, the results demonstrated that the varied scanning speed and UV per se has insignificant effect on cell viability but great impact on printing resolution.

**Fig 5:**
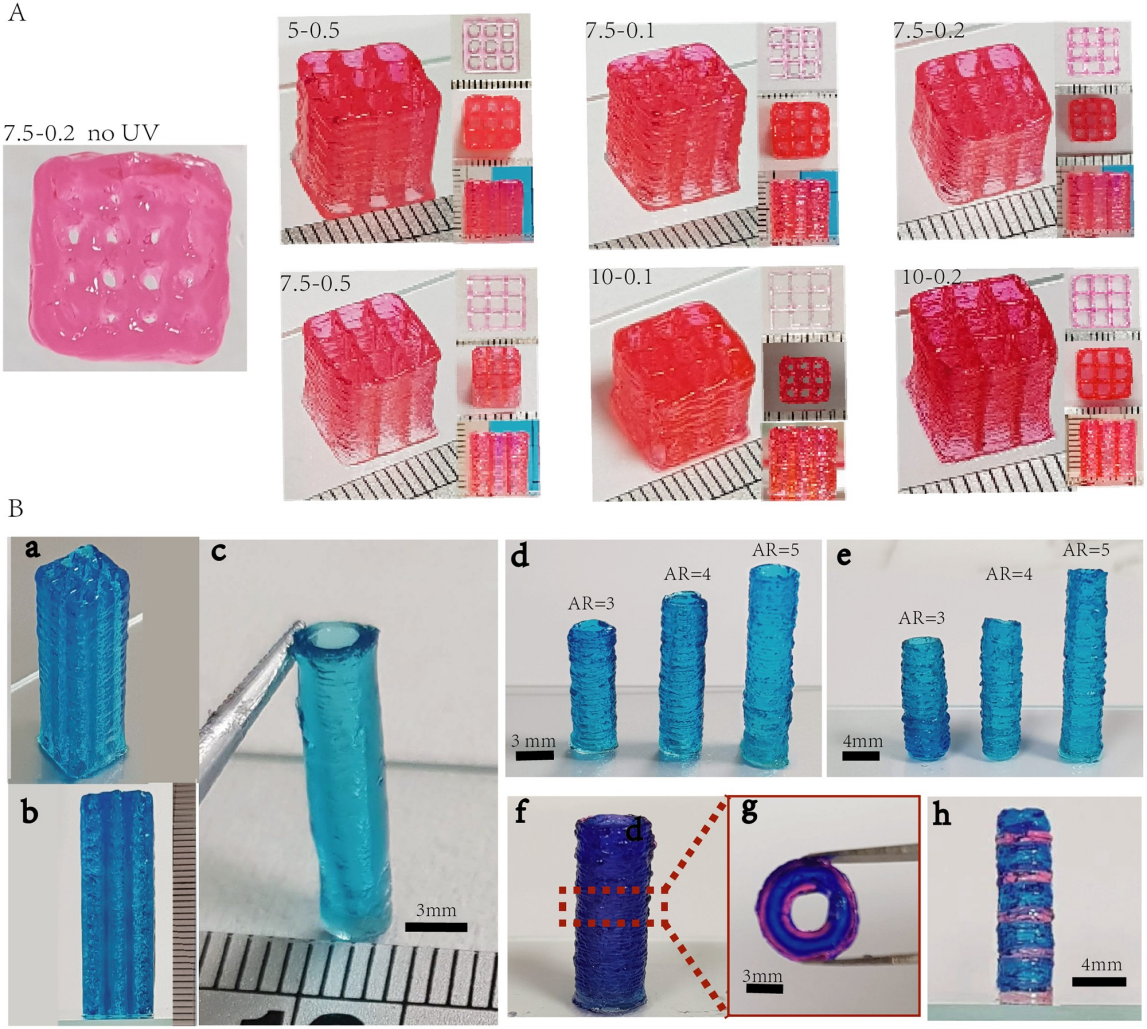
A) Left: Printed grid construct with no layer-by-layer UV curing using 7.5-0.2 group. Right: Printed grid pattern (W× L× H = 9mm × 9mm × 10mm) with the 6 selected GelMA-GG bio-inks. B) a. Printed grid construct (W× L × H = 9mm × 9mm × 30mm). b. Side view of the printed construct (W × L × H = 9mm × 9mm × 30mm. c-e. Tubular structures printed with GelMA-GG bio-ink (7.5-0.2) with different AR which is bioprintable and cell permissive. f-h. Multiple materials deposition with the layer-by-layer UV curing strategy.

Upon establishing the effects of UV scanning speed on cell viability and printing resolution, 3D grid and tubular constructs were printed to demonstrate the ability to print complex structures with high aspect ratio (AR) using the proposed layer-by-layer UV-assisted bioprinting approach (printing speed 100mm/min, UV scanning speed 400mm/min) (Fig 5). Both grid pattern (W× L = 9mm × 9mm) with a height of 10mm (Fig 5A) and a height of 30mm (Fig 5Ba and 5Bb) can be printed with all the selected groups of materials using the layer-by-layer UV curing strategy. Conversely, printing without using the layer-by-layer UV curing method yielded constructs with poor resolution and building up taller structures were challenging. Furthermore, it is to be noted that the difficulty of printing high aspect ratio structures increases as the diameter of the printed constructs decreases. The smallest tubular structure that can be successfully printed with high repeatability is of 3mm diameter with a high AR of 5 (as shown in Fig 5Bd). As a proof-of-concept, multi-material printing was performed in the transverse and longitudinal directions to demonstrate the ability to print multi-material constructs with high aspect ratio (Fig 5Bf, 5Bg and 5Bh). Firstly, multi-material printing in the transverse direction was demonstrated by fabricating concentric tubular structures of different diameters in Fig 5Bf and 5Bg. Next, multi-material printing in the longitudinal direction was demonstrated by fabricating tubular structures of different distinct regions as shown in Fig 5Bh. As clearly demonstrated by the printed grid and tubular structures, the layer-by-layer UV curing strategy has shown great improvement in constructing taller structures (AR≥5) and better resolution than those printed with post curing strategy.

### Post-printing phase

#### Material property (mechanical stiffness and microstructure)

**Fig 6:**
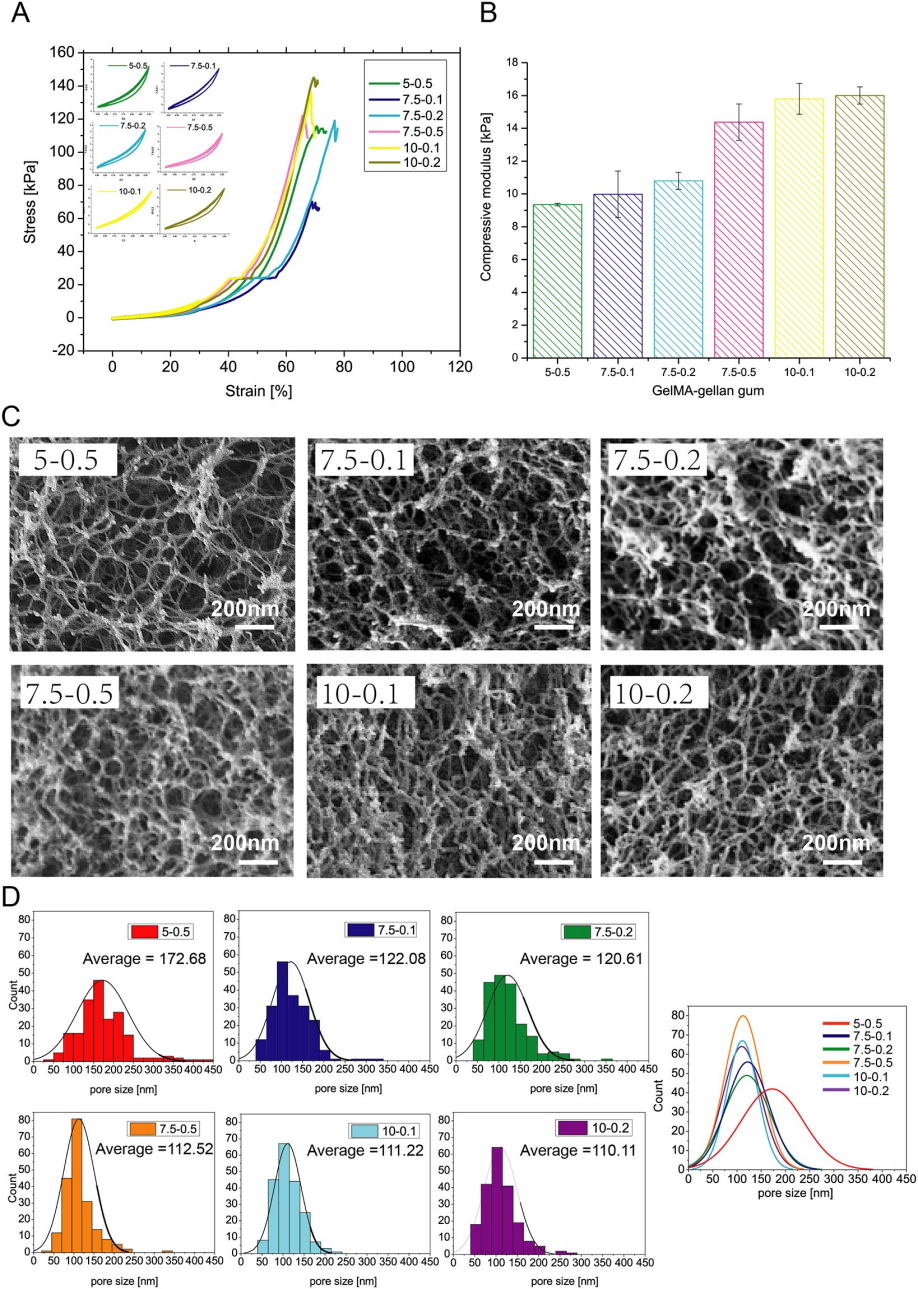
Bio-ink properties (mechanical stiffness and microstructure). A) Cyclic compression test. B) Compressive modulus of GelMA-GG bio-inks with different concentrations. C) FE-SEM imaging of GelMA-GG with varied concentrations; scale bar = 200 nm. D) Pore size distribution of GelMA-GG hydrogel bio-inks.

The matrix stiffness is one of the critical factors that regulate cell behaviors; therefore, it is crucial to consider the matrix stiffness of the printing material when designing such in vitro tissue constructs ([53, 54]). The material stiffness influences cell migration, differentiation and proliferation. Driven by this, we have measured the material stiffness of the selected composite hydrogel with good printability (5-0.5%, 7.5-0.1%, 7.5-0.2%, 7.5-0.5%, 10-0.1%, 10-0.2% w/v). These printed constructs were subjected to 5 times pre-cyclic under 30% strain. The cyclic compression curves of all the groups exhibited similar cyclic recovery performance, which indicated excellent recovery capability of the bio-inks (Fig 6A). As revealed by Fig 6B, the composite bio-inks exhibited a large range of compressive modulus. The results suggest that higher polymer concentration leads to higher compressive modulus, since a range of compressive modulus from 9kPa to 16kPa could be achieved by tuning the polymer concentration. It is important to note that the modulus could be further adjusted by UV intensity and exposure time. With the increased UV intensity and exposure time, the hydrogel stiffness can be increased to the scale of a hundred kPa ([55]).

Further in-depth characterization of the material stiffness was performed by evaluating the micro-structure of the bio-inks using Field-emission scanning electron microscope (FE-SEM), as shown in Fig 6C. The different printed GelMA-GG bio-ink constructs were subjected to critical point drying to carefully preserve the native micro-structure found in the GelMA-GG bio-ink. FE-SEM imaging of the different bio-inks at 30,000x magnifications indicated a porous 3D microenvironment and highly-interconnected pores within the GelMA-GG printed constructs. ImageJ was used to analyse the FE-SEM images to determine the pore sizes and porosity within the GelMA-GG microstructures at varying concentrations. The FE-SEM images reveal that a 5-0.5% w/v GelMA-GG printed construct showed the largest pore size (172.7 ± 63.9 nm), whereas 10-0.2% w/v GelMA-GG printed construct showed the smallest pore size (110.1 ± 38.9 nm). The pore size of the 3D GelMA-GG printed constructs generally decreased with increasing polymer concentration but the influence of GelMA concentration is more significant than that of GG. For a constant 2.5% increase in GelMA concentration; a significant 34.8% reduction in pore size was observed when comparing between 5-0.5% w/v (172.7 ± 63.9 nm pore size) and 7.5-0.5% w/v (112.5 ± 37.9 nm pore size), whereas only a small reduction of 1.2% in pore size was observed when comparing between 7.5-0.1% w/v (122.1 ± 43.8 nm pore size) and 7.5-0.2% w/v (120.6 ± 47.3 nm pore size). Further change in GG concentration did not result in significant changes in pore size (6.7% difference between 7.5-0.2% w/v and 7.5-0.5% w/v). In conclusion, pore size is shown to be more dramatically affected by GelMA than GG concentration with pore size decreasing with increase of polymer concentration as summarized in Table 1. The pore size of the constructs lies between 110 and 170nm, which is relatively small for cells to migrate. Therefore, it is crucial to control the degradation rate of the GelMA-GG construct. It is reported that gellan gum and GelMA degrade quite slowly in PBS [56]. However, gellan gum and GelMA could be gradually degraded in presence of lysozyme and collagenase, respectively. As demonstrated in the work by Xu et al. [56], gellan gum degraded to 20% of their initial weight after 16 days when immersed in PBS with 0.5 mg/mL lysozyme. Degradation of pure GelMA is concentration dependent. It is demonstrated that GelMA with a concentration lower than 10% can be totally degraded within 5days [57]. Taken together, we infer that the degradation of GelMA-GG construct in vivo is concentration dependent and therefore could be well modulated to facilitate cell migration and ingrowth.

**Table 1:** Porosity and average pore size of GelMA-GG with different concentrations.

	Porosity (%)	Average pore size (nm)
5-0.5	65.33	172.7 ± 63.9
7.5-0.1	53.08	122.1 ± 43.89
7.5-0.2	52.17	120.6 ± 47.3
7.5-0.5	43.11	112.5 ± 37.9
10-0.1	42.38	111.2 ± 31.8
10-0.2	41.11	110.1 ± 38.9

#### Influence of bio-ink on cells: Manual casting of cell-laden bio-inks

**Fig 7:**
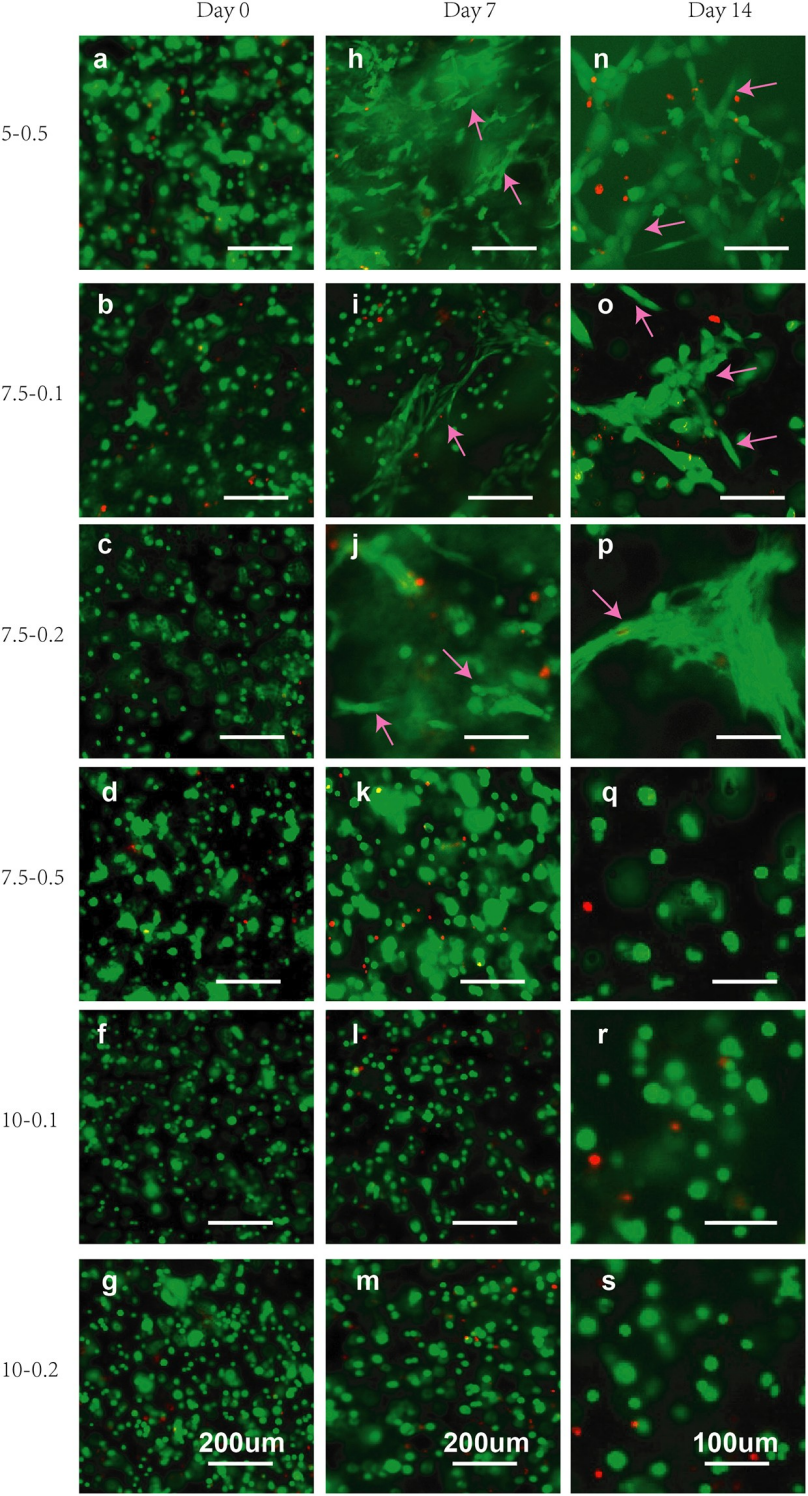
Live/dead staining of C2C12 in manual-cast cell-laden bio-inks with varied concentrations on Days 0, 7 and 14, with pink arrows showing cell elongation and spreading.

To evaluate the biocompatibility of the hydrogel blends, C2C12 cells were encapsulated inside the 3D GelMA-GG constructs over a period of 14-day culture. Live/dead staining was used to characterize the cell behavior at Days 0, 7 and 14. The presence of living cells (≥ 95% of total encapsulated cells) within all the composite GelMA-GG constructs indicated high cell viability at all time points (Day 0, 7 and 14) as shown in Fig 7. Furthermore, cell elongation and spreading (as depicted in Fig 7) in 5-0.5%, 7.5-0.1% and 7.5-0.2% w/v GelMA-GG constructs on Day 7 indicated that the bio-ink micro-structure and stiffness were supportive of C2C12 differentiation. Notably, 5-0.5% w/v GelMA-GG bio-ink showed a much better cell spreading and elongation than the 7.5-0.1 and 7.5-0.2% w/v GelMA-GG bio-inks. In contrast, the C2C12 cells in the 7.5-0.5, 10-0.1 and 10-0.2% w/v GelMA-GG bio-inks mostly remained round in shape within the 3D bio-ink constructs. The results demonstrated that pore size and mechanical stiffness of the bio-ink have critical influence in regulating cell behavior.

#### Influence of bio-ink on cells: 3D bioprinting of cell-laden bio-inks

**Fig 8:**
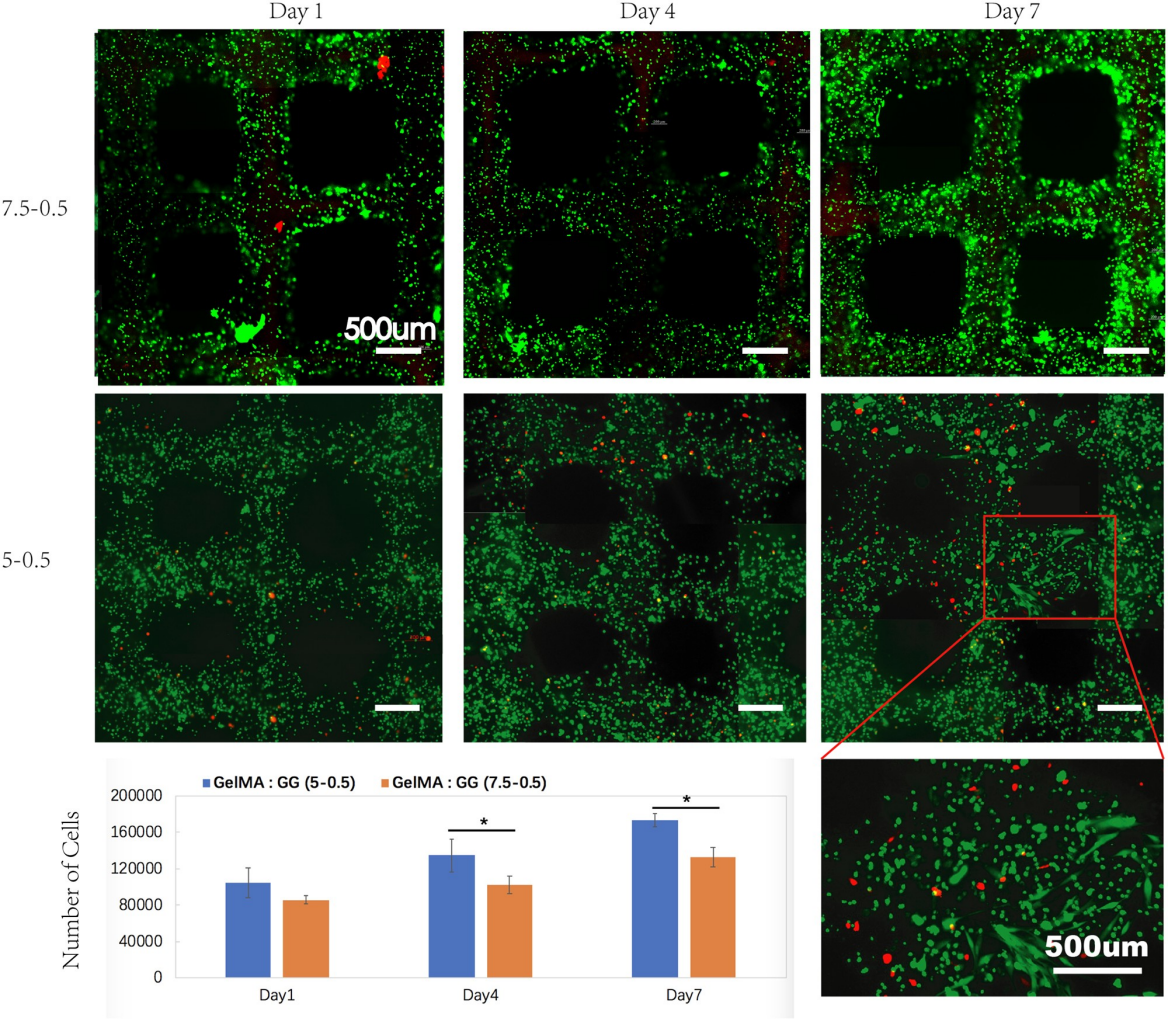
C2C12 cell viability and proliferation study of cell printing on Day 1,4 and 7; scale bar is 500 *μ*m. *—p < 0.05, **—p < 0.001.

By performing the manual casting approach, we can easily select the suitable composite bio-inks for our desired tissue engineering applications (specifically for soft tissue engineering in this work). The C2C12 cells in 5-0.5%, 7.5-0.1% and 7.5-0.2% w/v GelMA-GG constructs were able to spread and proliferate well due to the more favorable material micro-structures and stiffness, whereas the C2C12 cells in 7.5-0.5%, 10-0.1% and 10-0.2% w/v GelMA-GG constructs remained round. To understand whether the results in the manual-casting approach could be reproduced in the printed constructs, we chose 1 type of bio-ink from each group (elongated cells in the 5-0.5% w/v GelMA-GG constructs and round cells in the 7.5-0.5% w/v GelMA-GG constructs) for further experiments. The C2C12 cells were printed using the 5-0.5% and 7.5-0.5% w/v GelMA-GG bio-inks into 3D lattice structure. The cell viability was evaluated at Days 1, 4 and 7 post-printing, as shown in Fig 8. The high viability of printed cells indicated that the bioprinting process had insignificant effect on the viability of cells. Similarly, cell elongation was clearly observed in 5-0.5% GelMA-GG bioprinted constructs on Day 7. The cell proliferation was characterized using a PrestoBlue assay by measuring the relative fluorescence units and comparing with the standardized cell density curve in Fig 8. Overall, the printed C2C12 cells in 5-0.5% w/v GelMA-GG bioprinted constructs showed a faster proliferation rate relative to the printed C2C12 cells in the 7.5-0.5% w/v GelMA-GG bioprinted constructs as indicated by the faster increase in cell number over 7 days of study (Fig 8). The results were in good agreement with the manual-casting study, which proved that our bioprinting process does not have adverse effects on cells survival and differentiation.

## Conclusion

The GelMA-based bio-inks have exhibited great biocompatibility for cells due to the presence of RGD peptides. However, challenges in using GelMA as bio-ink remains especially in printability and poor structure integrity. In this work, we have presented a layer-by-layer UV-assisted bioprinting strategy to fabricate complex 3D bioprinted constructs with high aspect ratio for tissue engineering of soft tissues using the GelMA-GG bio-inks. To strike a balance between printability and biocompatibility, minimum ideal amount of gellan gum was added to enforce the printability of the bioinks without compromising the biocompatibility. In-depth characterization and evaluation on the different composite GelMA-GG bio-inks were performed to select a suitable range of GelMA-GG bio-inks through our proposed parametric study. The three main phases of bio-ink development involve 1) bio-ink preparation phase, 2) printing phase and 3) post-printing phase. From our work, a suitable range of bio-ink viscosity lower than 0.124 Pa·s at 37°C was found to be suitable for cell encapsulation and to achieve a homogeneous cell-laden bio-inks. Material viscosity of 0.2-1.0 Pa·s at a printing temperature of 25°C is recommended for printing of complex 3D cell-laden constructs with high aspect ratio using our layer-by-layer UV-assisted bioprinting strategy. The critical role of UV source in printing process has been investigated, specifically, the UV influence on printing resolution and cell survival rate. In addition, a strong correlation between material microstructure and stiffness has been shown in our study and their synergistic influence on cell behavior has been investigated. In conclusion, this work has presented an effective approach to fabricate complex 3D structures with great structure integrity, high aspect ratio, good shape fidelity and mechanical stability of soft materials and in this case GelMA-GG composite bio-ink. This method could be easily adapted for all light curable materials and would find great potential in scaffold/bioprinting for tissue engineering of soft tissues.

## References

[1] Chua CK, Yeong WY. Bioprinting: principles and applications. vol. 1 World Scientific Publishing Co Inc; 2014.

[2] Chua CK, Leong KF. 3D Printing and Additive Manufacturing: Principles and Applications (with Companion Media Pack) of Rapid Prototyping Fifth Edition World Scientific Publishing Company; 2017.

[3] Ng WL, Wang S, Yeong WY, Naing MW. Skin bioprinting: impending reality or fantasy? Trends in Biotechnology. 2016;34(9):689–699. 10.1016/j.tibtech.2016.04.006 27167724

[4] Zhuang P, Sun AX, An J, Chua CK, Chew SY. 3D neural tissue models: From spheroids to bioprinting. Biomaterials. 2018;154:113–133. 10.1016/j.biomaterials.2017.10.002 29120815

[5] Ng WL, Qi JTZ, Yeong WY, Naing MW. Proof-of-concept: 3D bioprinting of pigmented human skin constructs. Biofabrication. 2018;10(2):025005 10.1088/1758-5090/aa9e1e 29360631

[6] Bhuthalingam R, Lim PQ, Irvine SA, Agrawal A, Mhaisalkar PS, An J, et al A novel 3D printing method for cell alignment and differentiation. International Journal of Bioprinting. 2015;1(1):57–65.

[7] Mehrban N, Teoh GZ, Birchall MA. 3D bioprinting for tissue engineering: Stem cells in hydrogels. International journal of bioprinting. 2016;2(1).

[8] Ng WL, Goh MH, Yeong WY, Naing MW. Applying macromolecular crowding to 3D bioprinting: fabrication of 3D hierarchical porous collagen-based hydrogel constructs. Biomaterials science. 2018;6(3):562–574. 10.1039/c7bm01015j 29383354

[9] Rice JJ, Martino MM, De Laporte L, Tortelli F, Briquez PS, Hubbell JA. Engineering the regenerative microenvironment with biomaterials. Advanced healthcare materials. 2013;2(1):57–71. 10.1002/adhm.201200197 23184739

[10] Huang G, Wang L, Wang S, Han Y, Wu J, Zhang Q, et al Engineering three-dimensional cell mechanical microenvironment with hydrogels. Biofabrication. 2012;4(4):042001 10.1088/1758-5082/4/4/042001 23164720

[11] Yu H, Tay CY, Pal M, Leong WS, Li H, Li H, et al A Bio-inspired Platform to Modulate Myogenic Differentiation of Human Mesenchymal Stem Cells Through Focal Adhesion Regulation. Advanced healthcare materials. 2013;2(3):442–449. 10.1002/adhm.201200142 23184715

[12] Tay CY, Koh CG, Tan NS, Leong DT, Tan LP. Mechanoregulation of stem cell fate via micro-/nano-scale manipulation for regenerative medicine. Nanomedicine. 2013;8(4):623–638. 10.2217/nnm.13.31 23560412

[13] Hsieh FY, Lin HH, Hsu Sh. 3D bioprinting of neural stem cell-laden thermoresponsive biodegradable polyurethane hydrogel and potential in central nervous system repair. Biomaterials. 2015;71:48–57. 10.1016/j.biomaterials.2015.08.028 26318816

[14] Pati F, Jang J, Ha DH, Kim SW, Rhie JW, Shim JH, et al Printing three-dimensional tissue analogues with decellularized extracellular matrix bioink. Nature communications. 2014;5:3935 10.1038/ncomms4935 24887553 PMC4059935

[15] Kang HW, Lee SJ, Ko IK, Kengla C, Yoo JJ, Atala A. A 3D bioprinting system to produce human-scale tissue constructs with structural integrity. Nature biotechnology. 2016;34(3):312 10.1038/nbt.3413 26878319

[16] Suntornnond R, Tan EYS, An J, Chua CK. A mathematical model on the resolution of extrusion bioprinting for the development of new bioinks. Materials. 2016;9(9):756 10.3390/ma9090756 PMC5457067 28773879

[17] Ozbolat IT, Hospodiuk M. Current advances and future perspectives in extrusion-based bioprinting. Biomaterials. 2016;76:321–343. 10.1016/j.biomaterials.2015.10.076 26561931

[18] Ng WL, Yeong WY, Naing MW. Polyelectrolyte gelatin-chitosan hydrogel optimized for 3D bioprinting in skin tissue engineering. International Journal of Bioprinting. 2016;2(1).

[19] Ng WL, Yeong WY, Naing MW. Potential of bioprinted films for skin tissue engineering. 2014.

[20] Saunders RE, Derby B. Inkjet printing biomaterials for tissue engineering: bioprinting. International Materials Reviews. 2014;59(8):430–448. 10.1179/1743280414Y.0000000040

[21] Tse CCW, Ng SS, Stringer J, MacNeil S, Haycock JW, Smith PJ. Utilising inkjet printed paraffin wax for cell patterning applications. 2016.

[22] Ng WL, Lee JM, Yeong WY, Naing MW. Microvalve-based bioprinting–process, bio-inks and applications. Biomaterials science. 2017;5(4):632–647. 10.1039/c6bm00861e 28198902

[23] Ng WL, Yeong WY, Naing MW. Polyvinylpyrrolidone-based bio-ink improves cell viability and homogeneity during drop-on-demand printing. Materials. 2017;10(2):190 10.3390/ma10020190 PMC5459162 28772551

[24] Koch L, Brandt O, Deiwick A, Chichkov BN. Laser-assisted bioprinting at different wavelengths and pulse durations with a metal dynamic release layer: A parametric study. International Journal of Bioprinting 3 (2017), Nr 1. 2017;3(1):42–53. 10.18063/IJB.2017.01.001 PMC7575628 33094176

[25] Lin H, Zhang D, Alexander PG, Yang G, Tan J, Cheng AWM, et al Application of visible light-based projection stereolithography for live cell-scaffold fabrication with designed architecture. Biomaterials. 2013;34(2):331–339. 10.1016/j.biomaterials.2012.09.048 23092861 PMC3612429

[26] Malda J, Visser J, Melchels FP, Jüngst T, Hennink WE, Dhert WJ, et al 25th anniversary article: engineering hydrogels for biofabrication. Advanced materials. 2013;25(36):5011–5028. 10.1002/adma.201302042 24038336

[27] Annabi N, Tamayol A, Uquillas JA, Akbari M, Bertassoni LE, Cha C, et al 25th anniversary article: Rational design and applications of hydrogels in regenerative medicine. Advanced materials. 2014;26(1):85–124. 10.1002/adma.201303233 24741694 PMC3925010

[28] Panwar A, Tan LP. Current status of bioinks for micro-extrusion-based 3D bioprinting. Molecules. 2016;21(6):685 10.3390/molecules21060685 PMC6273655 27231892

[29] Jang TS, Jung HD, Pan HM, Han WT, Chen S, Song J. 3D printing of hydrogel composite systems: Recent advances in technology for tissue engineering. International Journal of Bioprinting. 2018;4(1). 10.18063/ijb.v4i1.126 PMC7582009 33102909

[30] Bertlein S, Brown G, Lim KS, Jungst T, Boeck T, Blunk T, et al Thiol–ene clickable gelatin: a platform bioink for multiple 3D biofabrication technologies. Advanced Materials. 2017;29(44):1703404 10.1002/adma.201703404 29044686

[31] Naseer SM, Manbachi A, Samandari M, Walch P, Gao Y, Zhang YS, et al Surface acoustic waves induced micropatterning of cells in gelatin methacryloyl (GelMA) hydrogels. Biofabrication. 2017;9(1):015020 10.1088/1758-5090/aa585e 28195834 PMC5421404

[32] Wang Z, Tian Z, Menard F, Kim K. Comparative study of gelatin methacrylate hydrogels from different sources for biofabrication applications. Biofabrication. 2017;9(4):044101 10.1088/1758-5090/aa83cf 28770808

[33] Zhou M, Lee BH, Tan LP. A dual crosslinking strategy to tailor rheological properties of gelatin methacryloyl. International Journal of Bioprinting. 2017;3(2):130–137. 10.18063/IJB.2017.02.003 PMC7575630 33094187

[34] Yue K, Trujillo-de Santiago G, Alvarez MM, Tamayol A, Annabi N, Khademhosseini A. Synthesis, properties, and biomedical applications of gelatin methacryloyl (GelMA) hydrogels. Biomaterials. 2015;73:254–271. 10.1016/j.biomaterials.2015.08.045 26414409 PMC4610009

[35] Sensini A, Gualandi C, Cristofolini L, Tozzi G, Dicarlo M, Teti G, et al Biofabrication of bundles of poly (lactic acid)-collagen blends mimicking the fascicles of the human Achille tendon. Biofabrication. 2017;9(1):015025 10.1088/1758-5090/aa6204 28224971

[36] Stratesteffen H, Köpf M, Kreimendahl F, Blaeser A, Jockenhoevel S, Fischer H. GelMA-collagen blends enable drop-on-demand 3D printablility and promote angiogenesis. Biofabrication. 2017;9(4):045002 10.1088/1758-5090/aa857c 28795951

[37] Diamantides N, Wang L, Pruiksma T, Siemiatkoski J, Dugopolski C, Shortkroff S, et al Correlating rheological properties and printability of collagen bioinks: the effects of riboflavin photocrosslinking and pH. Biofabrication. 2017;9(3):034102 10.1088/1758-5090/aa780f 28677597

[38] Nichol JW, Koshy ST, Bae H, Hwang CM, Yamanlar S, Khademhosseini A. Cell-laden microengineered gelatin methacrylate hydrogels. Biomaterials. 2010;31(21):5536–5544. 10.1016/j.biomaterials.2010.03.064 20417964 PMC2878615

[39] Lee BH, Lum N, Seow LY, Lim PQ, Tan LP. Synthesis and characterization of types a and b gelatin methacryloyl for bioink applications. Materials. 2016;9(10):797 10.3390/ma9100797 PMC5456596 28773918

[40] Mouser VH, Melchels FP, Visser J, Dhert WJ, Gawlitta D, Malda J. Yield stress determines bioprintability of hydrogels based on gelatin-methacryloyl and gellan gum for cartilage bioprinting. Biofabrication. 2016;8(3):035003 10.1088/1758-5090/8/3/035003 27431733 PMC4954607

[41] Zhu W, Harris BT, Zhang LG. Gelatin methacrylamide hydrogel with graphene nanoplatelets for neural cell-laden 3D bioprinting. In: Engineering in Medicine and Biology Society (EMBC), 2016 IEEE 38th Annual International Conference of the. IEEE; 2016. p. 4185–4188. 10.1109/EMBC.2016.7591649 28269205

[42] Shin SR, Zihlmann C, Akbari M, Assawes P, Cheung L, Zhang K, et al Reduced graphene oxide-gelMA hybrid hydrogels as scaffolds for cardiac tissue engineering. Small. 2016;12(27):3677–3689. 10.1002/smll.201600178 27254107 PMC5201005

[43] Navaei A, Saini H, Christenson W, Sullivan RT, Ros R, Nikkhah M. Gold nanorod-incorporated gelatin-based conductive hydrogels for engineering cardiac tissue constructs. Acta biomaterialia. 2016;41:133–146. 10.1016/j.actbio.2016.05.027 27212425

[44] Ostrovidov S, Ahadian S, Ramon-Azcon J, Hosseini V, Fujie T, Parthiban SP, et al Three-dimensional co-culture of C2C12/PC12 cells improves skeletal muscle tissue formation and function. Journal of tissue engineering and regenerative medicine. 2017;11(2):582–595. 10.1002/term.1956 25393357

[45] Liu W, Heinrich MA, Zhou Y, Akpek A, Hu N, Liu X, et al Extrusion Bioprinting of Shear-Thinning Gelatin Methacryloyl Bioinks. Advanced healthcare materials. 2017;6(12):1601451 10.1002/adhm.201770062 PMC5545786 28464555

[46] Xavier JR, Thakur T, Desai P, Jaiswal MK, Sears N, Cosgriff-Hernandez E, et al Bioactive nanoengineered hydrogels for bone tissue engineering: a growth-factor-free approach. ACS nano. 2015;9(3):3109–3118. 10.1021/nn507488s 25674809

[47] Coutinho DF, Sant SV, Shin H, Oliveira JT, Gomes ME, Neves NM, et al Modified Gellan Gum hydrogels with tunable physical and mechanical properties. Biomaterials. 2010;31(29):7494–7502. 10.1016/j.biomaterials.2010.06.035 20663552 PMC2933815

[48] Melchels FP, Dhert WJ, Hutmacher DW, Malda J. Development and characterisation of a new bioink for additive tissue manufacturing. Journal of Materials Chemistry B. 2014;2(16):2282–2289. 10.1039/c3tb21280g 32261716

[49] Shin H, Olsen BD, Khademhosseini A. The mechanical properties and cytotoxicity of cell-laden double-network hydrogels based on photocrosslinkable gelatin and gellan gum biomacromolecules. Biomaterials. 2012;33(11):3143–3152. 10.1016/j.biomaterials.2011.12.050 22265786 PMC3282165

[50] Kazemzadeh-Narbat M, Rouwkema J, Annabi N, Cheng H, Ghaderi M, Cha BH, et al Engineering photocrosslinkable bicomponent hydrogel constructs for creating 3D vascularized bone. Advanced healthcare materials. 2017;6(10):1601122 10.1002/adhm.201601122 28240417

[51] Ouyang L, Yao R, Mao S, Chen X, Na J, Sun W. Three-dimensional bioprinting of embryonic stem cells directs highly uniform embryoid body formation. Biofabrication. 2015;7(4):044101 10.1088/1758-5090/7/4/044101 26531008

[52] Chang R, Nam J, Sun W. Effects of dispensing pressure and nozzle diameter on cell survival from solid freeform fabrication–based direct cell writing. Tissue Engineering Part A. 2008;14(1):41–48. 10.1089/ten.a.2007.0004 18333803

[53] Cavo M, Fato M, Peñuela L, Beltrame F, Raiteri R, Scaglione S. Microenvironment complexity and matrix stiffness regulate breast cancer cell activity in a 3D in vitro model. Scientific reports. 2016;6:35367 10.1038/srep35367 27734939 PMC5062115

[54] Lantoine J, Grevesse T, Villers A, Delhaye G, Mestdagh C, Versaevel M, et al Matrix stiffness modulates formation and activity of neuronal networks of controlled architectures. Biomaterials. 2016;89:14–24. 10.1016/j.biomaterials.2016.02.041 26946402

[55] Bartnikowski M, Wellard RM, Woodruff M, Klein T. Tailoring hydrogel viscoelasticity with physical and chemical crosslinking. Polymers. 2015;7(12):2650–2669. 10.3390/polym7121539

[56] Xu Z, Li Z, Jiang S, Bratlie KM. Chemically Modified Gellan Gum Hydrogels with Tunable Properties for Use as Tissue Engineering Scaffolds. ACS omega. 2018;3(6):6998–7007. 10.1021/acsomega.8b00683 30023967 PMC6044625

[57] Zhao X, Lang Q, Yildirimer L, Lin ZY, Cui W, Annabi N, et al Photocrosslinkable gelatin hydrogel for epidermal tissue engineering. Advanced healthcare materials. 2016;5(1):108–118. 10.1002/adhm.201500005 25880725 PMC4608855

